# Preclinical Application of Augmented Reality in Pediatric Craniofacial Surgery: An Accuracy Study

**DOI:** 10.3390/jcm12072693

**Published:** 2023-04-04

**Authors:** Federica Ruggiero, Laura Cercenelli, Nicolas Emiliani, Giovanni Badiali, Mirko Bevini, Mino Zucchelli, Emanuela Marcelli, Achille Tarsitano

**Affiliations:** 1 Department of Biomedical and Neuromotor Science, University of Bologna, 40138 Bologna, Italy; 2 Maxillo-Facial Surgery Unit, AUSL Bologna, 40124 Bologna, Italy; 3 Laboratory of Bioengineering—eDIMES Lab, Department of Medical and Surgical Sciences (DIMEC), University of Bologna, 40138 Bologna, Italy; 4 Oral and Maxillo-Facial Surgery Unit, IRCCS Azienda Ospedaliero-Universitaria di Bologna, Via Albertoni 15, 40138 Bologna, Italy; 5 Pediatric Neurosurgery, IRCCS Istituto delle Scienze Neurologiche di Bologna, Via Altura 3, 40138 Bologna, Italy

**Keywords:** augmented reality, craniofacial surgery, head and neck surgery, computer-assisted surgery

## Abstract

Background: Augmented reality (AR) allows the overlapping and integration of virtual information with the real environment. The camera of the AR device reads the object and integrates the virtual data. It has been widely applied to medical and surgical sciences in recent years and has the potential to enhance intraoperative navigation. Materials and methods: In this study, the authors aim to assess the accuracy of AR guidance when using the commercial HoloLens 2 head-mounted display (HMD) in pediatric craniofacial surgery. The Authors selected fronto-orbital remodeling (FOR) as the procedure to test (specifically, frontal osteotomy and nasal osteotomy were considered). Six people (three surgeons and three engineers) were recruited to perform the osteotomies on a 3D printed stereolithographic model under the guidance of AR. By means of calibrated CAD/CAM cutting guides with different grooves, the authors measured the accuracy of the osteotomies that were performed. We tested accuracy levels of ±1.5 mm, ±1 mm, and ±0.5 mm. Results: With the HoloLens 2, the majority of the individuals involved were able to successfully trace the trajectories of the frontal and nasal osteotomies with an accuracy level of ±1.5 mm. Additionally, 80% were able to achieve an accuracy level of ±1 mm when performing a nasal osteotomy, and 52% were able to achieve an accuracy level of ±1 mm when performing a frontal osteotomy, while 61% were able to achieve an accuracy level of ±0.5 mm when performing a nasal osteotomy, and 33% were able to achieve an accuracy level of ±0.5 mm when performing a frontal osteotomy. Conclusions: despite this being an in vitro study, the authors reported encouraging results for the prospective use of AR on actual patients.

## 1. Introduction

Augmented reality (AR) is a computerized technology which transfers virtual information to real environments. The technology is based on the “onlay” principle, i.e., the camera “reads” the object in the frame and the system recognizes it and activates a new level of communication, overlapping and integrating the virtual information with the actual object or environment [1]. Unlike Virtual Reality (VR), which creates a totally computer-generated artificial environment, AR uses the real environment and overlays new virtual information on top of it, thus providing a composite view that enhances the user’s sensory perception of the world. Tools and systems employing AR have been designed and tested in the context of several medical applications [2,3,4,5,6,7,8,9,10,11,12,13], including surgical navigation in neurosurgery [14], craniomaxillofacial surgery [1,15,16,17,18,19,20,21], and head and neck oncology [22].

In recent years, its use in surgical navigation has been widely validated; many procedures, such as ventriculo-peritoneal shunt insertion and tumor resection in craniofacial and neurosurgery, are now usually performed using AR navigation [14].

Standard surgical navigation relies on an external device that recognizes the patient’s position in the environment and then merges information from patient imaging, e.g., computer tomography (CT) or magnetic resonance imaging (MRI) on the screen.

The navigator ensures that the noble anatomical structures can be localized before and during the operation. This improves the safety of the procedure. By means of navigation, it is also possible to import onto the patient’s imaging the trajectories of osteotomies and the target lesions in order to improve the extension and invasiveness of the surgery itself [1,17].

Though conventional navigation is a well-established methodology in surgery, it is burdened by several drawbacks. One of these is the fact that the operator has to switch his or her attention continuously between the patient and the screen [1,12,15].

AR head-mounted displays (HMDs) represent a technology that overcomes this limitation and can improve surgical navigation. Indeed, HMDs have integrated displays in order to allow surgeons to receive pertinent information while focusing their view on the surgical field [12]. HMDs enable the operator to visualize noble structures, trajectories for osteotomies, and incision points which are directly superimposed onto the patient. In optical see-through HMDs, such as the HoloLens 2 (Microsoft), the natural sight of the surgeon is not compromised since the holograms are projected on transparent lenses.

Craniofacial surgery is a subspecialty that aims to address congenital skull dysmorphologies in pediatric patients. Among these, we cite single-suture craniosynostosis, multiple-suture synostosis, and syndromic craniosynostosis.

It is a surgery that demands a certain amount of accuracy to avoid noble structures. Additionally, the morphological surgical outcome relies on the accurate design of the osteotomy [23].

In this study, the authors aim to assess the accuracy of AR guidance when using the commercial HoloLens 2 HMD [24] in pediatric craniofacial surgery.

In particular, we focus on the procedure used to address frontal skull anomalies, known as fronto-orbital remodeling (FOR).

While this is a pre-clinical study on a 3D printed phantom, the authors ultimately want to demonstrate the extent to which AR guidance using the HoloLens 2 smart glasses can accurately reproduce osteotomy trajectories in pediatric craniofacial surgery.

## 2. Materials and Methods

This study was designed in order first to implement the AR-based protocol using the HoloLens 2 smart glasses. The authors then arranged a test session to evaluate the success rate in executing the AR-guided osteotomies for fronto-orbital remodeling (FOR) on a 3D printed phantom.

In the following sections, the development phase and the experimental phase of the study are discussed.

### 2.1. Development Phase

#### 2.1.1. Virtual Content Preparation

**Figure 1:**
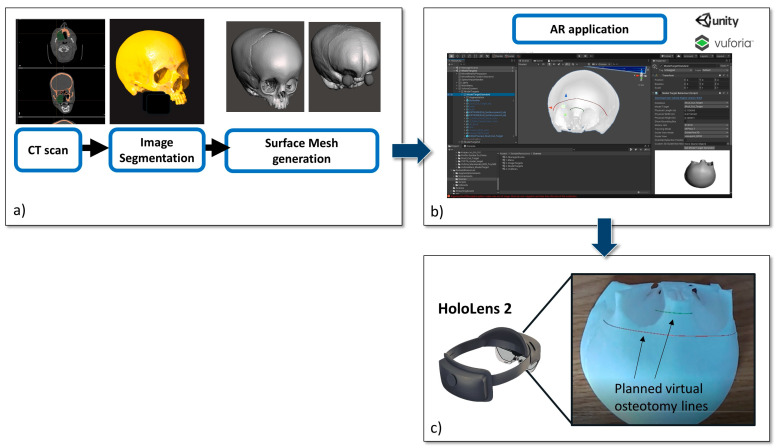
Development phase: (**a**) from CT scan to virtual content preparation; the virtual 3D skull model was also 3Dprinted to obtain a patient-specific phantom; (**b**) Unity software interface used for AR application development; (**c**) the planned osteotomy lines displayed in AR with HoloLens 2 smart glasses.

We selected the preoperative CT scan of a patient who had already been admitted and undergone an operation (study protocol CE 499-2022-OSS-AUSLBO). A DICOM file dataset was acquired and segmented in order to reconstruct a three-dimensional (3D) virtual model of the skull. Areas of the subject’s head that were of anatomical interest (e.g., bones, brain, eye globes, and skin) were segmented using Mimics (Materialise, Leuven, Belgium). Next, 3D meshes were generated from all the segmented masks and saved in standard tessellation language (STL) format (Figure 1).

#### 2.1.2. 3D Printing of Skull Phantom and CAD/CAM Templates for Testing Accuracy

An appropriate portion of the reconstructed skull was selected for printing. In particular, relying on clinical information, we decided to visualize the skull from the top, as it would be viewed in theatre, and to cut the model behind the coronal sutures and bilaterally at the level of the fronto-zygomatic sutures.

From the cut STL files, a phantom model made of photosensitive resin was produced by means of a stereolithography (SLA) 3D printer (Form 3, Formlabs, Somerville, MA, USA).

**Figure 2:**
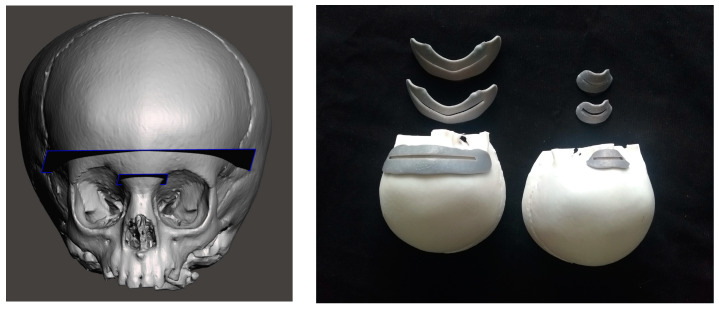
On the left, the planned osteotomies for the fronto-orbital bandeau of the Fronto Orbital Remodeling (FOR) on the right, the 3D printed cutting guides with calibrated grooves for both osteotomies.

To evaluate the AR guidance accuracy, CAD/CAM templates were designed using MeshMixer 3.5 software (Autodesk Inc., Mill Valley, CA, USA), and these were to be positioned on the surface of the phantom model, as cutting guides, in correspondence with the planned FOR osteotomies. For the AR-guided task, we selected the nasal and the frontal osteotomies that are part of the fronto orbital remodeling (Figure 2, left).

The templates were 3D printed (Form 3, Formlabs) with grooves of different widths (3 mm, 2 mm, 1 mm) in order to evaluate three levels of achievable accuracy (±1.5 mm, ±1.0 mm, and ±0.5 mm) (Figure 2, right). Strips of calibrated adhesive tape were applied to each template and used to measure the cumulative length of the traced osteotomy included within the grooves. We considered the AR-guided tasks successfully completed (100% success rate) when the traced osteotomy profile fell within the grooves of the cutting guides along their entire length (nasal osteotomy: 27 mm; frontal osteotomy: 75 mm).

#### 2.1.3. The AR Application

The virtual skull model with all its components (bone, skin, eye globes and brain), was imported into the Unity 3D software 2019.4.21f1 (Unity Technologies, San Francisco, CA, USA), extended with a specific software development kit for creating augmented reality apps (Vuforia Engine package 9.8.5, PTC, Inc., Boston, MA, USA).

By means of the Vuforia Engine software, the registration between the virtual osteotomy traces and the skull phantom was achieved using the “model target” function, which allows the system to recognize the shape of an actual object to be tracked. In order to achieve this, the object has to be observed from a certain perspective by the surgeon wearing the AR glasses. In this case, we decided to reproduce the point of view of the surgeon in the theatre when performing the surgical procedure for fronto-orbital remodeling. The application draws and projects in front of the user wearing the AR glasses a profile (“guide view”) of the model target; the user simply needs to move the lenses and match the projected drawing on the actual object.

In this study, a 3D model of the patient’s skull was used as the model target for the virtual-to-real scene registration.

The AR application generates several holograms, which are superimposed on the printed skull portion, for each structure we want to visualize, i.e., the bony skull, the skin, the brain, the eye globes, and the FOR osteotomy trajectories to be used as guiding information during the surgical task.

The AR application was built as a UWP (Universal Windows Platform) app deployed on the Microsoft HoloLens 2 smart glasses.

Interactive user interface toggles (check boxes) were added to turn the rendering of each virtual anatomical structure and the planned virtual osteotomy trajectories on and off. Voice commands to show/hide the virtual structures were also implemented in order to provide a completely hands-free AR guidance system.

### 2.2. Experimental Phase

The authors tested the AR application for the HoloLens 2 by having the selected FOR osteotomies, i.e., the nasal osteotomy (27 mm long) and the frontal osteotomy (75 mm long) performed under its guidance.

We recruited three surgeons and three engineers (three females and three males aged between 25 and 50 years). Each user repeated the procedure six times on the same 3D printed phantom with intervals of one week between each trial. Each user was briefed about the task that they were to perform. They had to perform the osteotomies on the phantom under the AR guidance provided by the HoloLens 2. Each user, after having calibrated the HMD for the perception of the optimal holograms, looked at the phantom in order to track it via the model target tracking function and then began the AR-guided task. Each user carefully drew the trajectory of the osteotomy on the skull phantom with a pencil, following the planned trajectories which were displayed as holograms in dashed lines. Vocal commands allowed the users to show/hide the virtual structures during the execution of their tasks.

Afterward, using the 3D printed templates for accuracy evaluation, another operator assessed the extent to which the line traced under AR guidance fell into the groove of the single template. Each template had a calibrated tape along the groove itself to facilitate the measurements.

### 2.3. Statistics

All measurements were recorded in an Excel spreadsheet file. Percentages were recorded and both a Kruskal–Wallis test and a Mann–Whitney test were performed on the measurements.

SPSS software (IBM, Armonk, New York, NY, USA) was used to perform the statistical analysis, and a *p* value of <0.05 was considered statistically significant. 

## 3. Results

The results are summarized in Table 1. With the HoloLens 2, 97% of the users were able to successfully trace the osteotomy trajectory with an accuracy level of ±1.5 mm (verified with the “3 mm” template) for the nasal cut. The percentage falls to 80% when looking at the frontal cut.

For accuracy levels of ±1 mm and ±0.5 mm, lower success rates were recorded. Specifically, for the nasal cut, we reported success rates of 80% and 61%, respectively. Regarding the frontal cut, the users were able to accomplish the task with an accuracy level ± 1 mm in 52% of cases, whereas only 33% precisely followed the groove with an accuracy level of ±0.5 mm.

The Kruskal–Wallis test demonstrated that all the users were able to complete the nasal cut task with no significant differences between them. However, more inter-operator differences were reported for the frontal cut (Table 2).

From the measurements, only one outlier was evident according to the Mann–Whitney test (Table 3).

The users reported that the usability of the AR guidance system was very good, but most of them reported a perceived loss of image quality when moving the pencil in front of the visor.

## 4. Discussion

AR is a promising technology in the medical field; more and more studies on its applications, especially in the surgical field, are being published due to increasing interest [1,2,10,11,12,13,14,15,16,17,25,26,27,28,29].

Its introduction enables the realization of a full concept of navigation, i.e., the operator does not have to shift his or her attention anymore and can stay focused on the patient, on whom holograms are projected. A specific taxonomy was introduced in the 1960s to categorize this technology [13].

AR HMDs are divided into optical see-through devices and video see-through devices [11,12,13,15,16,17,23,24,25,26,27,28,29,30,31]. In this study, the authors used the HoloLens 2, an optical see-through HMD.

Craniofacial surgery, and in particular corrective procedures for forehead morphological anomalies, can be challenging due to the noble structures underlying the bone [23]. Furthermore, accuracy in the execution of osteotomies is necessary in order to achieve a good result. In most cases of single-suture synostosis, the indication is merely morphological. Therefore, errors over a certain amount can lead to disastrous results. However, it has been explained in the literature that a 2 mm error is still acceptable and will not necessarily compromise the overall result [25,26]

Therefore, an HMD suitable for this surgery has to be accurate and maintain the hologram in the field of vision even if the surgeon moves. The HoloLens has already demonstrated good potential in this sense [13,24].

However, different drawbacks and limitations have been reported in the literature, such as depth of perception and registration errors [17,32].

We have reported some drawbacks, too. In an effort to overcome these limitations, our group has already tried to address the registration errors, in which a static error ranging from 1 mm to 10 mm is reported. This results in a misalignment in the subjective perception of the virtual image and actual image [17]. This registration error contributed to the overall error rate quantified in this study at the tracing stage, i.e., while performing AR-guided task of tracing the osteotomy lines on the skull phantom.

In this study, the authors evaluated the accuracy of the HoloLens 2 when used to perform a craniofacial surgery task. The procedure of choice was fronto-orbital remodeling, focusing in particular on two osteotomies, nasal and frontal, which define the orbital rim.

The authors selected six operators, and every operator had to repeat the task six times for each osteotomy, both nasal and frontal. In order to avoid an additive learning curve effect, an interval of one week passed between each trial.

The users were asked to trace the osteotomies with a pen under the guidance of the HoloLens 2 projection. They could see on the lenses the phantom and the dotted lines for the planned osteotomies. We noticed some errors in virtual-to-real alignment and a loss of sharpness when moving the pen in the field of view.

All the users were able to complete the task. The osteotomy traced with the pen was then checked with the cutting guides having different width grooves.

In terms of accuracy, our findings are consistent with previous findings outlined in the literature, and, to a certain extent, this is encouraging. We described the maximum accuracy with an error of ±1.5 mm. We also noticed that a lower level of accuracy was reported for the frontal cut, and this might be due to the length of the trace that had to be followed (75 mm), and to the more complex round anatomy.

Despite these technical pitfalls, the levels of accuracy that we reported are consistent with what has been described elsewhere in the literature [12,13,33,34,35].

Scherl et al. reported an accuracy of less than 1.3 mm in their in vivo study [13], whereas Tang et al., in their account of their experience with the HoloLens 2 in head and neck oncology, reported that the mean deviation between the preoperative virtual osteotomy plane and the actual postoperative osteotomy plane was 1.68 ± 0.92 mm, with the largest deviation being 3.46 mm [36].

Han et al. described their experience in craniosynostosis, and this is the only available on-patient work of its kind in the literature. Their study involved seven patients undergoing calvarial remodeling for plagiocephaly, and they compared the planned intracranial volume with the obtained one, with encouraging results [37]. In our study, we assessed another type of osteotomy for calvarial remodeling. This specific procedure consists of plain osteotomies and very limited areas for drilling and/or cutting. In this case, the authors focused on the simplest issues due to the technical limitations of the HoloLens 2. These included, but were not limited to, registration errors and the small augmentable field of view. Therefore, whereas there is a more stringent requirement for accuracy (i.e., sub-millimetric precision), other “surgery-specific” devices should be addressed, such as the ones previously described in the literature [17,38,39,40,41].

Our findings have, however, been encouraging. Our results included only one outlier, and therefore we cannot exclude a learning curve effect.

Our study had limitations, principally due to the advantageous lighting conditions compared with those typically found in theatre, the errors in model target registration, and operator-dependent factors. The authors also wish to emphasize that, despite the fact that loupe glasses are in common use, HMD technology has not yet been optimized for compatibility with loupes. Furthermore, the procedures were performed in ideal conditions. Our next step will be to implement the navigation system by inserting more details and more 3D objects, to be seen contemporaneously with the trajectories that are to be followed with the instruments (i.e., osteotomies). The preparation time for the 3D model reconstruction, starting from the DICOM data segmentation and including the setting up of the AR guidance software, was only one to two hours [17]. An in vivo study would be necessary to confirm these preliminary data.

## 5. Conclusions

This was an in vitro study. The encouraging results in terms of the ±1.5 mm accuracy make it suitable for application in craniofacial surgery, where such a margin of error can be admitted in certain tasks. More studies, including in vivo evaluations, are required to overcome the technical pitfalls involved in this promising technology.

## Figures and Tables

**Table 1:** Measurements taken from each recruited user. The left column shows the measurements for the nasal cut and the right column shows the measurements for the frontal cut. CG: cutting guide.

Nose				Frontal			
PT 1	CG (3 mm)	CG 2 (mm)	CG 1 (mm)	PT 1	CG (3 mm)	CG 2 (mm)	CG 1 (mm)
1	27 mm	27 mm	24 mm	1	75 mm	70 mm	40 mm
2	27 mm	27 mm	27 mm	2	75 mm	75 mm	55 mm
3	27 mm	25 mm	23 mm	3	75 mm	75 mm	60 mm
4	27 mm	24 mm	22 mm	4	75 mm	72 mm	65 mm
5	27 mm	27 mm	27 mm	5	70 mm	70 mm	60 mm
6	27 mm	26 mm	25 mm	6	75 mm	75 mm	75 mm
PT 2				PT 2			
1	27 mm	26 mm	23 mm	1	75 mm	55 mm	35 mm
2	27 mm	27 mm	26 mm	2	75 mm	75 mm	75 mm
3	27 mm	27 mm	27 mm	3	75 mm	73 mm	55 mm
4	27 mm	27 mm	27 mm	4	75 mm	75 mm	74 mm
5	27 mm	27 mm	25 mm	5	75 mm	75 mm	75 mm
6	27 mm	27 mm	27 mm	6	75 mm	75 mm	35 mm
PT 3				PT 3			
1	27 mm	25 mm	20 mm	1	75 mm	75 mm	70 mm
2	27 mm	27 mm	27 mm	2	75 mm	71 mm	75 mm
3	27 mm	27 mm	27 mm	3	75 mm	75 mm	70 mm
4	27 mm	27 mm	27 mm	4	75 mm	75 mm	75 mm
5	27 mm	27 mm	27 mm	5	75 mm	70 mm	60 mm
6	27 mm	27 mm	27 mm	6	75 mm	70 mm	35 mm
PT 4				PT 4			
1	27 mm	27 mm	27 mm	1	75 mm	75 mm	65 mm
2	27 mm	15 mm	10 mm	2	75 mm	75 mm	75 mm
3	27 mm	27 mm	26 mm	3	75 mm	75 mm	75 mm
4	27 mm	27 mm	22 mm	4	75 mm	70 mm	55 mm
5	27 mm	27 mm	27 mm	5	73 mm	71 mm	65 mm
6	27 mm	27 mm	27 mm	6	70 mm	60 mm	60 mm
PT 5				PT 5			
1	27 mm	27 mm	27 mm	1	70 mm	50 mm	35 mm
2	27 mm	27 mm	27 mm	2	70 mm	65 mm	55 mm
3	20 mm	12 mm	11 mm	3	65 mm	45 mm	35 mm
4	27 mm	25 mm	25 mm	4	75 mm	59 mm	54 mm
5	27 mm	27 mm	27 mm	5	75 mm	75 mm	45 mm
6	27 mm	27 mm	27 mm	6	75 mm	57 mm	45 mm
PT 6				PT 6			
1	27 mm	27 mm	27 mm	1	75 mm	75 mm	75 mm
2	27 mm	27 mm	27 mm	2	65 mm	60 mm	55 mm
3	27 mm	27 mm	27 mm	3	75 mm	75 mm	75 mm
4	27 mm	27 mm	27 mm	4	75 mm	75 mm	75 mm
5	27 mm	27 mm	27 mm	5	75 mm	75 mm	75 mm
6	27 mm	27 mm	27 mm	6	75 mm	75 mm	75 mm

**Table 2:** Kruskal–Wallis Test demonstrating no significative differences between operators.

	fro 3 mm	fro 2 mm	fro 1 mm	nos 3 mm	nos 2 mm	nos 1 mm
KruskaI-WaIIis H	6.992	9.579	13.083	5.000	4.883	6.521
df	5	5	5	5	5	5
Asymp. Sig.	0.221	0.088	0.023	0.416	0.43	0.259

**Table 3:** Mann–Whitney test, according to which only one outlier between the operators was evident.

	op2–op1	op3–op1	op4–op1	op5–op1	op6–op1	op3–op2	op4–op2	op5–op2	op6–op2	op4–op3	op5–op3	op6–op3	op5–op4	op6–op4	op6–op5
Z	−0.315	−0.677	−1.051	−2.023	−1.841	0.921	−1.214	−1.786	−1.361	−0.412	−1.997	−0.984	−2.207	−0.816	−2.041
Asymp. Sig. (2-tailed)	0.752	0.498	0.293	0.043	0.066	0.357	0.225	0.074	0.174	0.68	0.046	0.343	0.027	0.414	0.041

## Data Availability

Data are available upon reasonable request.
